# Phosphoproteomics uncovers a neuroimmune perspective on trigeminal neuralgia: sexually dimorphic regulatory networks linking calcium channels to the complement cascade

**DOI:** 10.3389/fimmu.2026.1676019

**Published:** 2026-03-03

**Authors:** Xiaojie Zhai, Xianghong Lin, Linlin Zhang, Yuxuan Ren, He Miao, Mengmeng Miao, Yijun Chen, Yiwei Zhang, Changshun Huang

**Affiliations:** 1 Anesthesiology Department, The First Affiliated Hospital of Ningbo University, Ningbo, China; 2 Health Science Center, Ningbo University, Ningbo, Zhejiang, China

**Keywords:** complement C1q, MAPK-HSPB1 axis, neuroimmune interaction, phosphoproteomics, sexual dimorphism, trigeminal neuralgia

## Abstract

**Background:**

Trigeminal neuralgia (TN) is a neuropathic pain disorder with a marked female predominance. While transcriptional changes in TN are documented, the translational and post-translational landscapes—specifically protein abundance and phosphorylation states—within the trigeminal ganglion (TG) remain largely unexplored. Understanding these layers is essential to deciphering the mechanisms behind the disease’s sexual dimorphism.

**Methods:**

we utilized the chronic infraorbital nerve ligation (CION) method via an intraoral approach model in male and female Sprague-Dawley rats. Mechanical allodynia was confirmed via behavioral testing. On postoperative day 7, trigeminal ganglia were harvested to capture the somatic molecular response. We performed an integrated analysis using TMT-based quantitative proteomics and phosphoproteomics. Bioinformatics tools were employed to map differentially expressed proteins (DEPs), kinase-substrate relationships, and protein-protein interaction (PPI) networks.

**Results:**

The study quantified 5,820 proteins and 8,830 phosphopeptides. (1) A striking divergence was observed in pathological pathways. Females exhibited a robust neuroimmune signature characterized by the specific upregulation of complement components (C1QA, C1QC) and Kininogen (KNG1). In contrast, males showed alterations primarily in lipid metabolism and synaptic vesicle cycles. (2) Phosphoproteomics identified the MAPK signaling pathway as a shared mechanism. Specifically, phosphorylation of HSPB1 at Ser86 (pS86-HSPB1)—a target of the MAPKAPK2/3 cascade—was significantly elevated, linking stress signaling to cytoskeletal reorganization. (3) PPI analysis highlighted voltage-gated calcium channel subunits as central hubs connecting these sex-specific modules, validating the relevance of calcium channel modulation in TN management.

**Conclusions:**

This study presents the first dual-omics atlas of the injured trigeminal ganglion. We identify a female-specific “Complement-Kininogen” axis and a conserved “MAPK-HSPB1” phosphorylation pathway as key drivers of TN. These findings provide a molecular explanation for the clinical gender bias and suggest that therapeutic strategies may need to be sex-stratified, with complement inhibition holding particular potential for female patients.

## 1 Introduction

Trigeminal neuralgia (TN) is a chronic neuropathic pain syndrome characterized by paroxysms of severe, unilateral facial pain manifesting as stabbing or electric shock-like sensations. This pathology typically results from lesions affecting one or more divisions of the trigeminal nerve—ophthalmic, maxillary, or mandibular—responsible for facial sensory transmission (1). In classical TN, neurovascular compression at the nerve root entry zone represents the central pathophysiological mechanism, driving morphological remodeling and focal demyelination of primary afferent fibers (2). Acknowledged as a severely debilitating condition, TN profoundly impairs patient quality of life.

Despite significant research progress in recent decades, the pathophysiology of TN remains incompletely understood, and current clinical interventions frequently fail to achieve sustained disease control (3–5). TN is clinically stratified into three distinct etiologies: classical (attributed to neurovascular compression), secondary (arising from underlying pathologies such as multiple sclerosis or tumors), and idiopathic. These subtypes display divergent pathological features, contributing to heterogeneity in clinical presentation. Management strategies prioritize reducing the frequency and severity of paroxysms to restore functional quality of life. Pharmacotherapy serves as the first-line treatment, while surgical intervention is reserved for patients exhibiting drug resistance or intolerance (6). Epidemiologically, TN demonstrates a marked female predisposition and an age-dependent increase in incidence (7). Consequently, elucidating the molecular mechanisms underlying this sexual dimorphism remains a critical research priority.

Recent investigations have increasingly targeted the trigeminal ganglion to identify molecular mechanisms suitable for therapeutic intervention in TN (8). Despite these efforts, clinically viable therapeutic strategies derived from these molecular targets remain elusive. This gap highlights an urgent need for advanced preclinical studies to elucidate the specific molecular drivers of TN and identify novel targets for more effective management.

Protein phosphorylation, a fundamental post-translational modification (PTM), plays a pivotal role in the pathogenesis of neuropathic pain. As a ubiquitous regulatory mechanism governing cellular signal transduction (9), phosphorylation is dynamically controlled by the interplay between kinases and phosphatases to modulate neuronal excitability, central sensitization, and neuroplasticity (10). Within trigeminal nociceptive pathways, these signaling networks—particularly those involving ERK-dependent mechanisms—are critical for sensory processing and the establishment of distinct pain phenotypes (11). Furthermore, while the complement component C1q has been implicated in pain initiation and maintenance, its specific function within the peripheral nervous system remains poorly characterized (12). Consequently, the precise contribution of neuroimmune interactions to nociceptive modulation warrants rigorous investigation.

Although mass spectrometry-based phosphoproteomics has successfully mapped thousands of phosphorylation sites associated with human pathology (13), a critical knowledge gap persists. Specifically, the upstream kinase regulatory networks governing more than 90% of these phosphorylation events in the context of TN remain uncharacterized.

Therefore, the present study utilized a modified intraoral nerve ligation approach to establish a rat model of TN and comprehensively profile phosphorylation dynamics within the trigeminal ganglion. By integrating phosphoproteomics, we aimed to identify critical phosphorylation events and elucidate their mechanistic links to neuronal hyperexcitability and nociceptive phenotypes. This investigation addresses a critical gap in current knowledge and provides novel insights that may facilitate the development of targeted therapeutic strategies for TN.

## 2 Materials and methods

### 2.1 Animal preparation

Seven-week-old male and female Sprague-Dawley (SD) rats, weighing approximately 200–250 g, were purchased from the Laboratory Animal Center of Ningbo University. Rats were housed in cages under controlled conditions: temperature maintained at 23 ± 1 °C and a standard 12-h light/dark cycle. Standard laboratory chow and water were provided ad libitum. All rats underwent a 7-day acclimatization period prior to the commencement of any experimental procedures. All procedures were conducted in accordance with the guidelines established by the International Association for the Study of Pain (IASP) and were approved by the Animal Ethics Committee of Ningbo University (Approval No. AEWC-NBU20230304).

### 2.2 Trigeminal neuralgia rat model

#### 2.2.1 ​Intraoral approach

This method was modified from previously described protocols (14, 15). Briefly, rats were anesthetized via intraperitoneal injection of 3% sodium pentobarbital (60 mg/kg) and secured in a supine position. The mouth was held open. A ∼1 cm incision was made along the buccal gingival sulcus near the left maxillary first molar. A glass probe was used to carefully dissect and expose the left infraorbital nerve. Two ligatures of 5–0 absorbable chromic catgut suture were tied with moderate tension around the exposed infraorbital nerve, spaced approximately 2 mm apart. Sham-operated controls underwent identical exposure of the nerve without subsequent ligation. All surgical procedures were performed under aseptic conditions.

#### 2.2.2 Euthanasia

At the end of the experiment, all rats were euthanized via intraperitoneal injection of an overdose of 3% sodium pentobarbital (100 mg/kg). Following confirmation of deep anesthesia, transcardial perfusion with saline and 4% paraformaldehyde was performed, and the trigeminal neuralgia (TN) were subsequently harvested for further experiments.

### 2.3 Behavioral test

Prior to Von Frey testing, animals were allowed to acclimate to the testing environment for at least 30 minutes per day over a minimum period of three days. Individual rats were placed in customized testing cages, and a series of calibrated Von Frey filaments (ranging from 2, 4, 6, 7, 10, to 15 grams) were applied with gentle force to the cutaneous region innervated by the trigeminal nerve V2 branch. Filaments were tested in ascending order of force intensity, starting from the smallest.

A positive response was defined by meeting one or more of the following criteria:

1)Avoidance behaviors: Rapid retreating, turning, or curling movements;2)Facial grooming or spasm: Repetitive scratching of the stimulated facial area (≥2 times) or asymmetric facial twitching;3)Attack response: Immediate biting or snapping at the stimulus. Each filament was applied five times with a 30-second interval between applications. The mechanical withdrawal threshold was defined as the minimal force (in grams) eliciting at least three positive responses in five consecutive applications.

### 2.4 Proteomics sample preparation

#### 2.4.1 Protein extraction

Trigeminal ganglia with liquid nitrogen into cell powder and then transferred to a 5-mL centrifuge tube. After that, four volumes of lysis buffer (8 M urea, 1% protease inhibitor cocktail) was added to the cell powder, followed by sonication three minutes on ice using a high intensity ultrasonic processor (Scientz). (Note: For PTM experiments, inhibitors were also added to the lysis buffer, e.g. 3 μM TSA and 50 mM NAM for acetylation, 1% phosphatase inhibitor for phosphorylation). The remaining debris was removed by centrifugation at 12,000 g at 4 °C for 10 min. Finally, the supernatant was collected and the protein concentration was determined with BCA kit according to the manufacturer’s instructions.

#### 2.4.2 Trypsin digestion

The sample was slowly added to the final concentration of 20% (m/v) TCA to precipitate protein, then vortexed to mix and incubated for 2 h at 4 °C. The precipitate was collected by centrifugation at 4500 g for 5 min at 4 °C. The precipitated protein was washed with pre-cooled acetone for 3 times and dried for 1 min. The protein sample was then redissolved in 200 mM TEAB and ultrasonically dispersed. Trypsin was added at 1:50 trypsin-to-protein mass ratio for the first digestion overnight. The sample was reduced with 5 mM dithiothreitol for 30 min at 56 °C and alkylated with 11 mM iodoacetamide for 15 min at room temperature in darkness. Finally, the peptides were desalted by Strata X SPE column.

#### 2.4.3 Affinity enrichment

The DIA phosphorylated proteomics has an additional affinity enrichment step compared to proteomics. Peptide mixtures were first incubated with IMAC microspheres suspension with vibration in loading buffer (50% acetonitrile/0.5% acetic acid). To remove the non-specifically adsorbed peptides, the IMAC microspheres were washed with 50% acetonitrile/0.5% acetic acid and 30% acetonitrile/0.1% trifluoroacetic acid, sequentially. To elute the enriched phosphopeptides, the elution buffer containing 10% NH4OH was added and the enriched phosphopeptides were eluted with vibration. The supernatant containing phosphopeptides was collected and lyophilized for LC-MS/MS analysis.

### 2.5 Proteomics analysis

#### 2.5.1 Mass spectrometer

The tryptic peptides were dissolved in solvent A, directly loaded onto a home-made reversed-phase analytical column (25-cm length, 100 μm i.d.). The mobile phase consisted of solvent A (0.1% formic acid, 2% acetonitrile/in water) and solvent B (0.1% formic acid in acetonitrile). Peptides were separated with following gradient: 0–16 min, 2%-22%B;16–22 min, 22%-35%B;22–26 min, 35%-90%B;26–30 min, 90%B, and all at a constant flow rate of 450 nl/minon a NanoElute UHPLC system (Bruker Daltonics). The peptides were subjected to capillary source followed by the timsTOF Pro mass spectrometry. The electrospray voltage applied was 1.7 kV. Precursors and fragments were analyzed at the TOF detector. The timsTOF Pro was operated in data independent parallel accumulation serial fragmentation (dia-PASEF) mode. The full MS scan was set as100-1700(MS/MS scan range) and 22PASEF(MS/MS mode)-MS/MS scans were acquired per cycle. The MS/MS scan range was set as 395–1395 and isolation window was set as20 m/z.

#### 2.5.2 Database search

Building the Spectral Library: The DDA data were processed using Spectronaut (v.18) software coupped with Pulsar search engine.Tandem mass spectra were searched against Rattus_norvegicus_10116_PR_20231121.fasta (47943 entries) concatenated with reverse decoy database. The max missing cleavages was set as 2. Carbamidomethyl on Cys was specified as fixed modification. Acetylation on protein N-terminal, oxidation on Met and phosphorylation(S/T) were specified as variable modifications. False discovery rate (FDR) of protein, peptide and PSM was adjusted to < 1%.The corresponding spectral library was imported into Spectronaut (v.18) software to predicts the retention time by nonlinear correction and searched against with DIA data.

### 2.6 Bioinformatics methods

#### 2.6.1 Annotation methods

The process of GO annotation involves using the eggnog-mapper software to extract GO IDs from the identified proteins based on the EggNOG database, and then performing functional classification annotation analysis on the proteins according to cellular components, molecular functions, and biological processes.

In the project data, protein structural domain annotation was performed on the identified proteins based on the Pfam database and the corresponding PfamScan tool.

The Kyoto Encyclopedia of Genes and Genomes (KEGG) integrates currently identify proteins through BLAST comparison (blastp, evalue ≤ 1e-4), for each sequence, the annotation is based on the top-scoring comparison result.

In view of this, we used PSORTb software to perform subcellular structure prediction analysis of the above four classes of proteins identified in prokaryotes.

EggNOG provides a more comprehensive classification of species and more homologous protein sequences, with phylogenetic tree construction and functional annotation for each homologous gene cluster.

#### 2.6.2 Functional enrichment

Fisher’s exact test was used to analyze the significance of functional enrichment of differentially expressed proteins (using the identified protein as the background). Functional terms with Fold enrichment>1.5 and P value <0.05 were considered as significant.

##### 2.6.2.1 Enrichment-based clustering

We first collated all the categories obtained after enrichment along with their P values, and then filtered for those categories which were at least enriched in one of the clusters with P value <0.05. This filtered P value matrix was transformed by the function x = −log10 (P value). These p values were then clustered by one-way hierarchical clustering (Euclidean distance, average linkage clustering) in Genesis. Cluster membership were visualized by a heat map using the “Heatmap” function from the “ComplexHeatmap” R-package.

##### 2.6.2.2 Protein-protein interaction network

STRING defines a metric called “confidence score” to define interaction confidence; we fetched all interactions that had a confidence score > 0.7 (high confidence). Interaction network form STRING was visualized in R package “visNetwork”.

### 2.7 Western blots

Western blotting was performed according to previously established protocols (16, 17). Briefly, trigeminal ganglion (TG) samples were homogenized in NP-40 lysis buffer (#P0013F, Beyotime) supplemented with a protease inhibitor cocktail (#P8340, Sigma-Aldrich, St. Louis, MO, USA). Total protein (15 μg per lane) was separated via 12% SDS-PAGE and transferred onto polyvinylidene difluoride (PVDF) membranes. Membranes were blocked with 5% non-fat milk for 2 h at room temperature, followed by overnight incubation with primary antibodies at 4 °C. Subsequently, membranes were incubated with the corresponding HRP-conjugated secondary antibodies for 2 h at room temperature. Protein bands were visualized using the ChemiDoc XRS+ System (Bio-Rad, Hercules, CA, USA), and densitometric analysis was performed using ImageLab™ Software (Version 6.1, Bio-Rad). Target protein expression was normalized to $\beta$-actin levels and expressed as a percentage relative to the control group.

The following primary antibodies were utilized: anti-C1QB (1:1,000; #M019076S, Abmart, Shanghai, China), anti-KNG1 (1:500; #A1670, ABclonal, Wuhan, China), anti-C1QC (1:1,000; #16889-1-AP, Proteintech, Wuhan, China), anti-HSP27 (1:1,000; #ab317707, Abcam), and anti-C1QA (1:1,000; #ab189922, Abcam). Mouse anti-$\beta$-actin polyclonal antibody (1:10,000; #GB15003, Servicebio, Wuhan, China) was used as the loading control. Immunodetection was performed using HRP-conjugated secondary antibodies: anti-mouse IgG (1:3,000; #GB23301, Servicebio) and anti-rabbit IgG (1:3,000; #GB23303, Servicebio).

### 2.8 Statistical analysis

#### 2.8.1 Protein quantitative analysis

Repeat three times or more: Take the ratio of the mean relative quantification values of the modification site in two sets of samples as the fold change (FC). For example, calculate the difference multiple of modification sites between sample group A and sample group B. The calculation formula is as follows: R represents the relative quantitative value of the modification site, i represents the sample, and k represents the modification site.

FCA/B,k=Mean(Rik,i∈A)/Mean(Rik,i∈B).

Based on the above differential analysis, when the P value < 0.05, a change in differential expression level >1.5 is considered a significant upregulation threshold, and a change level < 1/1.5 is considered a significant downregulation threshold.

All statistical analyses were performed using Independent-Samples T Test or Paired Student’s t-test (SPSS 16.0 version). Asterisks (∗) indicated statistically significant differences from the control group (∗P <0.05, ∗∗P < 0.01).

#### 2.8.2 Drawing software

https://www.ptm-biolab-css.com.cn/cloud/cloudTool

https://go.drugbank.com/

https://guolab.wchscu.cn/AnimalTFDB4/#/TFBS_Predict

https://jaspar.elixir.no/

#### 2.8.3 Prediction of protein kinase activity

Upload the MS file after mass spectrometry analysis to the kinase prediction tool. The GSEA method was used to predict the phosphokinase activity of the sample and comparison group, and the NES value obtained by enrichment analysis method was used as the kinase activity score to evaluate kinase activity. Based on the complex regulatory relationship between kinases and phosphorylation sites, a kinase regulatory network was constructed for each comparison group by screening out phosphokinases with significantly activated or inhibited activity and phosphorylation sites with significantly different expression levels.

#### 2.8.4 Prediction of transcription factor binding site

The promoter series of the target gene of rattus norvegicus was obtained through NCBI (https://www.ncbi.nlm.nih.gov/), the Predict TFBS tool of AnimalTFDB4 was opened to input the promoter to submit the results, the TFBS with the highest score was sorted and filtered in the Download file, the first five same loci were taken after the same operation of jaspar was opened, and the Frequency Matrix was downloaded. The default display is in JASPAR format for logo drawing.

#### 2.8.5 Drug annotation analysis

First, the receptor CACN1B that is significantly related to the disease screened in the proteome or modification group was converted into the Uniprot protein name, and then the corresponding DrugBank (1)(v5.1.12) drug name, type, FDA certification and other information were obtained in batches. Screening of targeted relationships between FDA-approved drugs and inhibitors/activators for Sankey diagram presentation.

## 3 Results

### 3.1 Establishment of a trigeminal neuralgia rat model via distal infraorbital nerve ligation

**Figure 1:**
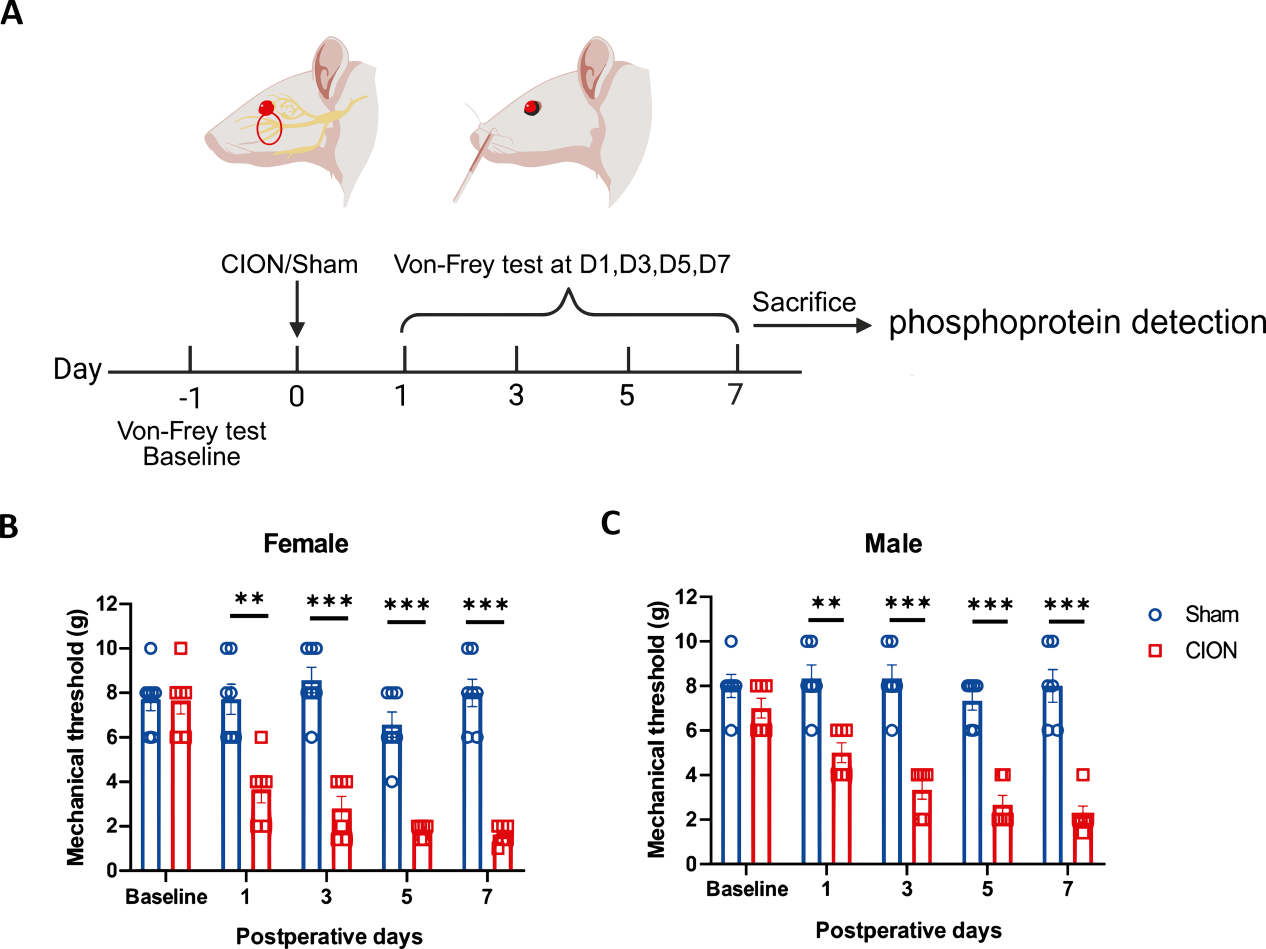
Chronic constriction of the infraorbital nerve induces mechanical hypersensitivity. **(A)** Schematic of the experimental design. Baseline behavioral assessments were conducted prior to chronic infraorbital nerve ligation (CION) or Sham surgery. Mechanical thresholds were evaluated on postoperative days (POD) 1, 3, 5, and 7, followed by tissue collection for RNA-Seq and phosphoproteomic analysis. **(B, C)** Assessment of mechanical thresholds in female **(B)** and male **(C)** rats. CION surgery significantly reduced facial mechanical withdrawal thresholds compared to Sham controls in both sexes. Data are presented as mean ± SEM (n = 6 per group). Statistical significance was determined using two-way ANOVA followed by Bonferroni’s *post hoc* test (** p < 0.01, *** p < 0.001 vs. Sham).

To establish a model of trigeminal neuralgia (TN), we utilized the chronic infraorbital nerve ligation (CION) method via an intraoral approach. Briefly, rats were anesthetized with 3% sodium pentobarbital. An incision was made at the gingival margin of the first maxillary molar to expose the infraorbital nerve (ION). Two loose ligatures were placed around the nerve using 5–0 absorbable sutures (**Figure 1A**). Sham-operated controls underwent identical nerve exposure without ligation. Mechanical sensitivity of the facial skin ipsilateral to the surgery was assessed using von Frey filaments on postoperative days (POD) 1, 3, 5, and 7. As shown in **Figures 1B, C**, rats subjected to CION exhibited significantly lower mechanical withdrawal thresholds compared to the Sham group across all tested time points (POD 1, p < 0.01; POD 3, 5, and 7, p < 0.001) in both female and male cohorts. The most pronounced hypersensitivity was observed on POD 7. Consequently, trigeminal ganglia (TG) harvested on POD 7 were selected for subsequent phosphoproteomic analysis.

### 3.2 Integrated proteomic and phosphoproteomic profiling of the rat trigeminal ganglion in trigeminal neuralgia

**Figure 2:**
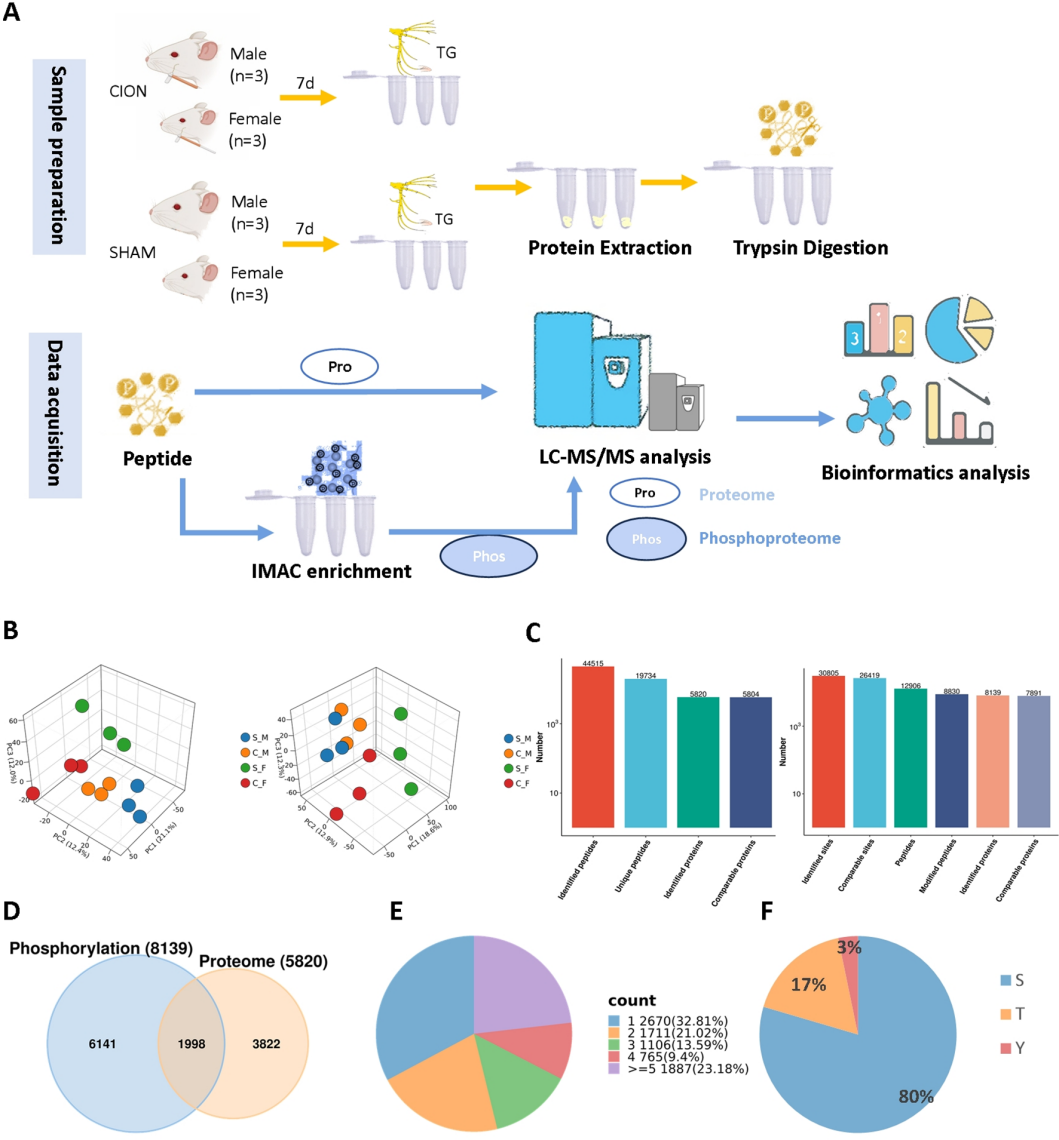
Global landscape of the trigeminal ganglion proteome and phosphoproteome in Trigeminal Neuralgia (TN). **(A)** Schematic workflow of the integrated proteomic and phosphoproteomic analysis. Tissues were processed for protein extraction, trypsin digestion, and immobilized metal affinity chromatography (IMAC) enrichment prior to LC-MS/MS. **(B)** Principal Component Analysis (PCA) of the proteomic (left) and phosphoproteomic (right) datasets. 3D scatter plots visualize the spatial distribution and reproducibility of samples from Sham (S_M/S_F) and CION (C_M/C_F) groups (n=3 per group). **(C)** Statistical summary of identified peptides, unique peptides, and proteins in the global proteome (left bar chart), and identified sites, peptides, and phosphoproteins in the phosphoproteome (right bar chart). **(D)** Venn diagram illustrating the overlap between proteins identified in the global proteome and the phosphoproteome. **(E)** Distribution of phosphorylation multiplicity. The pie chart displays the proportion of proteins containing 1, 2, 3, 4, or ge5 phosphorylation sites. **(F)** Distribution of phosphorylated amino acid residues: Serine (S), Threonine (T), and Tyrosine (Y).

To elucidate the molecular mechanisms underlying TN, we performed integrated liquid chromatography-tandem mass spectrometry (LC-MS/MS)-based proteomic and phosphoproteomic analyses on trigeminal ganglion (TG) tissues from CION-operated and Sham-operated rats of both sexes (**Figure 2A**). Principal component analysis (PCA) revealed distinct segregation between the TN model and Sham groups in both the global proteome and phosphoproteome datasets, indicating significant injury-induced molecular alterations (**Figure 2B**).

Stringent quality control criteria were applied to ensure high-confidence identifications: (1) false discovery rates (FDR) for both precursors and proteins were maintained below 1%, and (2) protein identification required at least one unique peptide. Based on these parameters, the global proteomic profiling yielded 19,734 unique peptides mapping to 5,820 proteins. In parallel, phosphoproteomic analysis identified 8,830 phosphopeptides corresponding to 8,139 phosphoproteins (**Figure 2C**). Comparative analysis indicated an overlap of 1,998 proteins detected in both datasets (**Figure 2D**).

Analysis of phosphorylation multiplicity revealed that 32.81% of the identified phosphoproteins (2,670 proteins) contained a single modification site. The majority of phosphoproteins were multi-phosphorylated, with 23.18% (1,887 proteins) harboring five or more phosphorylation sites (**Figure 2E**). Furthermore, the distribution of phosphorylated residues followed a canonical pattern, occurring predominantly on serine (S; 80%), followed by threonine (T; 17%) and tyrosine (Y; 3%) (**Figure 2F**).

### 3.3 Proteomic profiling reveals sex-specific and conserved molecular alterations in trigeminal neuralgia

**Figure 3:**
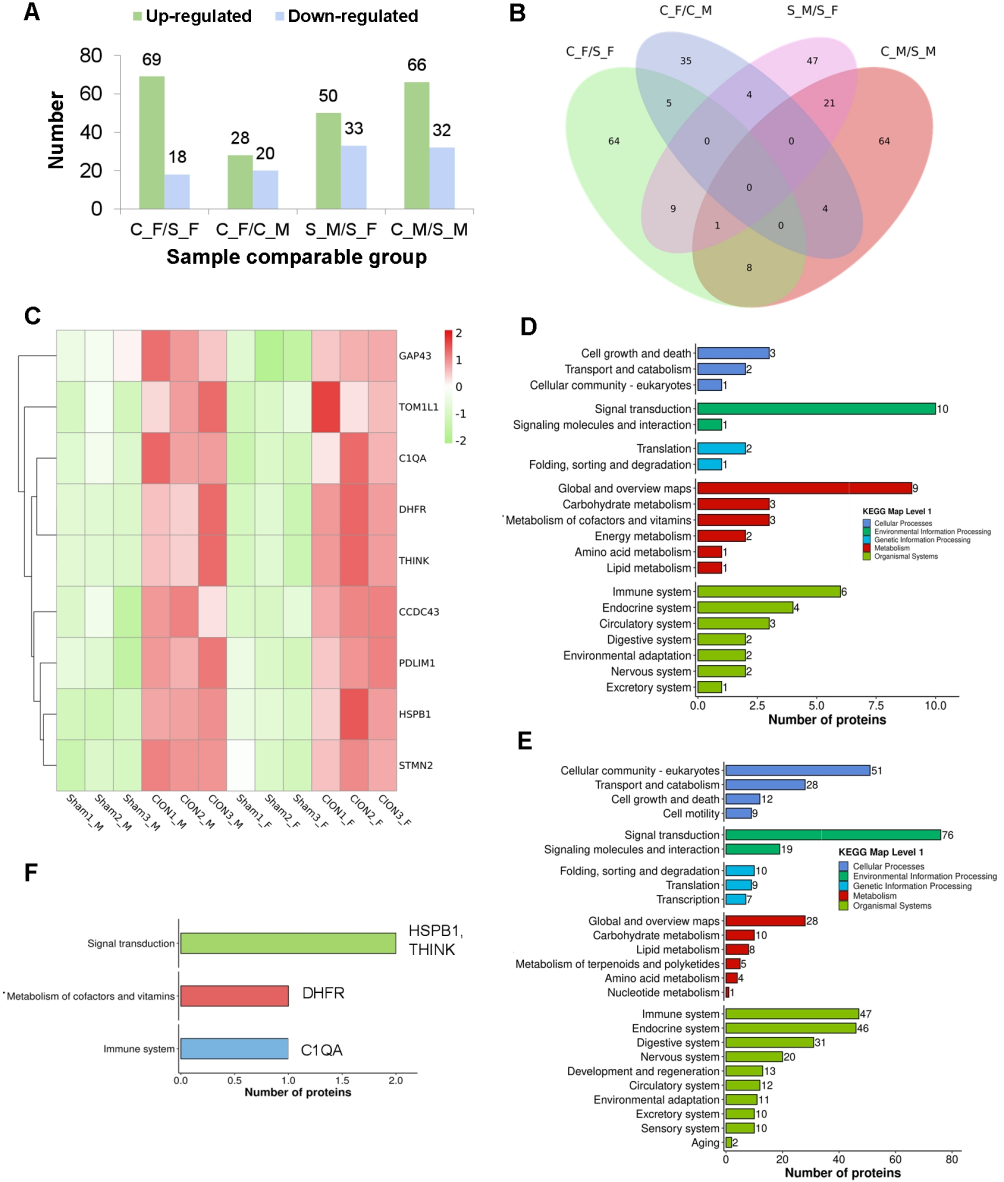
Differential proteomic expression and pathway analysis in Trigeminal Neuralgia. **(A)** Statistical summary of differentially expressed proteins (DEPs) across comparisons. The bar chart displays the count of upregulated (green) and downregulated (blue) proteins for each group comparison. **(B)** Venn diagram illustrating the overlap of DEPs between different comparison groups, highlighting nine proteins common to both male and female TN models. **(C)** Heatmap visualizing the expression levels of the nine common DEPs. The color scale (red to green) indicates the fold change in protein expression relative to the mean. **(D)** KEGG pathway classification analysis of DEPs identified in the TN model group (CION) versus the Sham group. **(E)** KEGG pathway classification analysis of DEPs identified between the Sham Male (S_M) and Sham Female (S_F) groups, reflecting baseline physiological differences. **(F)** Functional enrichment analysis of the nine common upregulated DEPs. Specific proteins are mapped to their primary KEGG categories: Signal transduction (HSPB1, THINK), Metabolism of cofactors and vitamins (DHFR), and Immune system (C1QA).

Following strict data filtering and normalization, we quantified differential protein expression profiles to identify molecular signatures associated with TN. Comparative analysis revealed 87 differentially expressed proteins (DEPs)—comprising 69 upregulated and 18 downregulated proteins—in the female TN model compared to sham controls (C_F vs. S_F). Similarly, 98 DEPs (66 upregulated, 32 downregulated) were identified in the male TN model compared to sham controls (C_M vs. S_M) (**Figure 3A**).Intersection analysis via a Venn diagram highlighted nine DEPs that were commonly modulated in both sexes (**Figure 3B**). Specifically, TOM1L1, PDLIM1, DHFR, THINK, HSPB1, C1QA, STMN2, CCDC43, and GAP43 were significantly upregulated in the CION groups of both sexes relative to their respective controls, suggesting a conserved core mechanism driving TN pathology (**Figure 3C**).

To elucidate the biological functions of these molecular changes, we performed Kyoto Encyclopedia of Genes and Genomes (KEGG) pathway enrichment analysis. DEPs identified in the CION groups were predominantly enriched in signal transduction and immune system pathways (**Figure 3D**). Interestingly, an analysis of baseline sexual dimorphism between sham-operated males and females (S_M vs. S_F) revealed that physiological sex differences are also heavily concentrated in these same pathways (**Figure 3E**).Finally, targeted KEGG analysis of the nine conserved upregulated proteins revealed distinct functional clusters: C1QA was enriched in immune system-related pathways (map04610), THINK and HSPB1 were enriched in signal transduction pathways (map04010 and map04013), and DHFR was associated with the metabolism of cofactors and vitamins (**Figure 3F**).

### 3.4 Dysregulation of the complement and coagulation cascades pathway in trigeminal neuralgia

**Figure 4:**
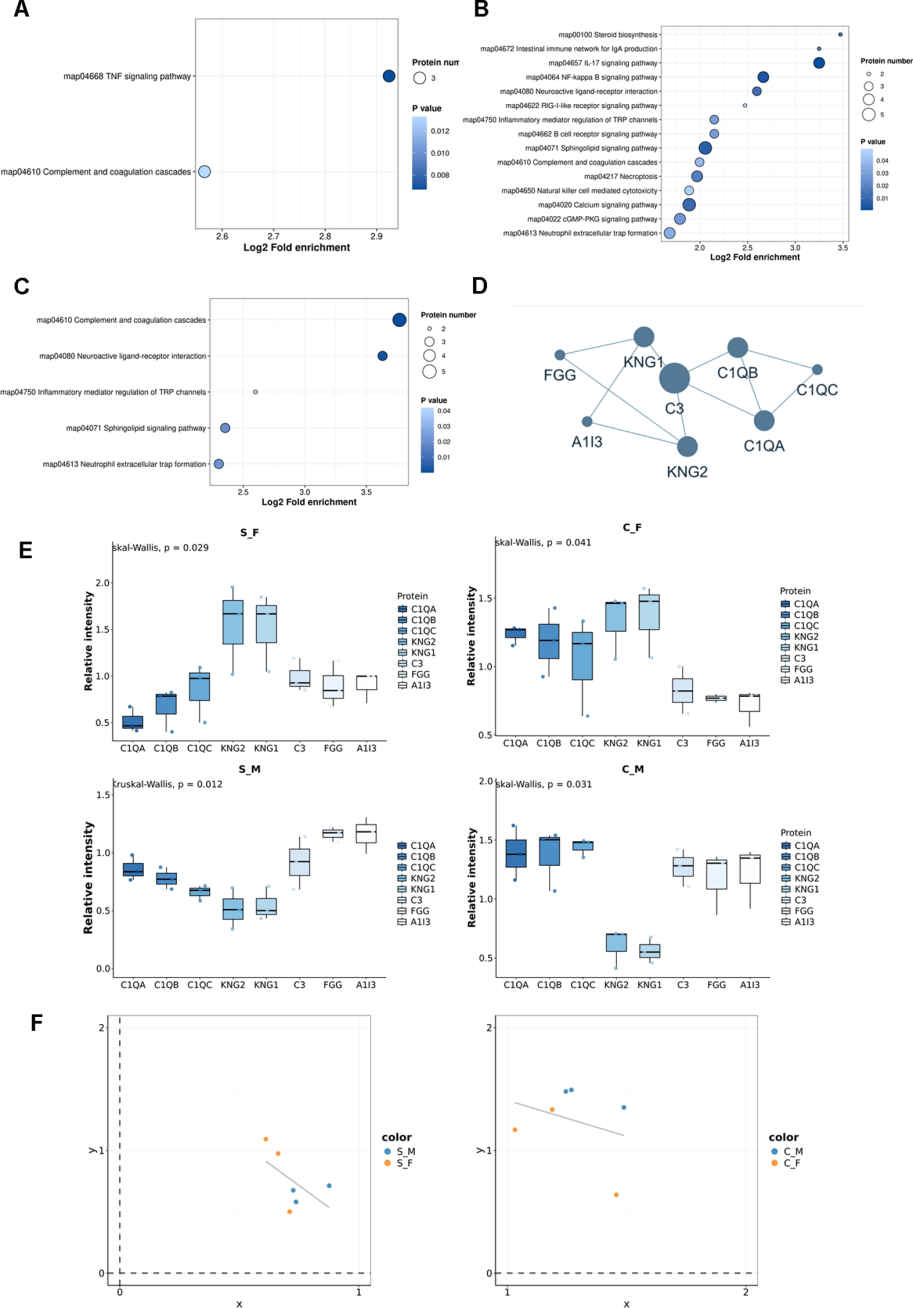
Alterations in the complement and coagulation cascades pathway associated with trigeminal neuralgia and sexual dimorphism. **(A)** KEGG pathway enrichment analysis of differentially expressed proteins (DEPs) between CION (TN model) and Sham groups. The “Complement and coagulation cascades” pathway (map04610) is significantly enriched. **(B)** KEGG pathway enrichment analysis of DEPs between Sham Male (S_M) and Sham Female (S_F) groups, highlighting baseline sex differences. **(C)** KEGG pathway enrichment analysis of DEPs between CION Female (C_F) and CION Male (C_M) groups. **(D)** Protein-protein interaction (PPI) network of DEPs mapped to the Complement and coagulation cascades pathway. Node size reflects connectivity. **(E)** Box plots displaying the relative expression intensity of key pathway proteins across groups (S_F, S_M, C_F, C_M). The central line represents the median, box limits indicate the upper and lower quartiles, and whiskers extend to the minimum and maximum values. Statistical significance among groups was determined using the Kruskal-Wallis test (p-values indicated in plots). **(F)** Scatter plots and linear regression analysis depicting the correlation between the relative intensities of C1QA (x-axis) and C1QC (y-axis). The negative slope indicates an inverse relationship between C1QA and C1QC abundance in both the Sham (left) and CION (right) cohorts.

To characterize specific molecular pathway alterations driving TN, we performed KEGG enrichment analysis on DEPs identified in the CION versus Sham comparison. Notably, the Complement and coagulation cascades pathway (map04610) was significantly enriched (**Figure 4A**). Interestingly, this pathway was also significantly enriched in comparisons reflecting sexual dimorphism, both in the baseline state (S_M vs. S_F; **Figure 4B**) and the disease state (C_F vs. C_M; **Figure 4C**), suggesting a convergence of disease-induced and sex-specific mechanisms. To elucidate the specific protein alterations within this pathway, we constructed a protein-protein interaction (PPI) network (**Figure 4D**) and analyzed the relative abundance of key constituents (**Figure 4E**). The analysis revealed distinct expression patterns for the Kininogen (KNG) and Complement C1q families. KNG1 expression was generally lower in TN models (C_M, C_F) compared to their respective Sham controls, yet maintained a sex-specific pattern with consistently higher expression in females than in males, independent of disease status. Conversely, the C1q family proteins (C1QA, C1QB, C1QC) exhibited marked upregulation in the TN model groups compared to Sham controls. Specifically, C1QA and C1QB levels were elevated in CION rats relative to Sham rats. Furthermore, baseline sexual dimorphism was evident, with C1QC expression being significantly higher in S_F compared to S_M (**Figure 4E**). These findings corroborate our earlier identification of C1QA as a key driver of pathway enrichment (Section 3.3). Finally, correlation analysis demonstrated a negative correlation between the relative expression levels of C1QA and C1QC across both sexes, suggesting a potential regulatory interplay (**Figure 4F**).

### 3.5 Validation of sex-dimorphic complement pathway proteins in trigeminal neuralgia

**Figure 5:**
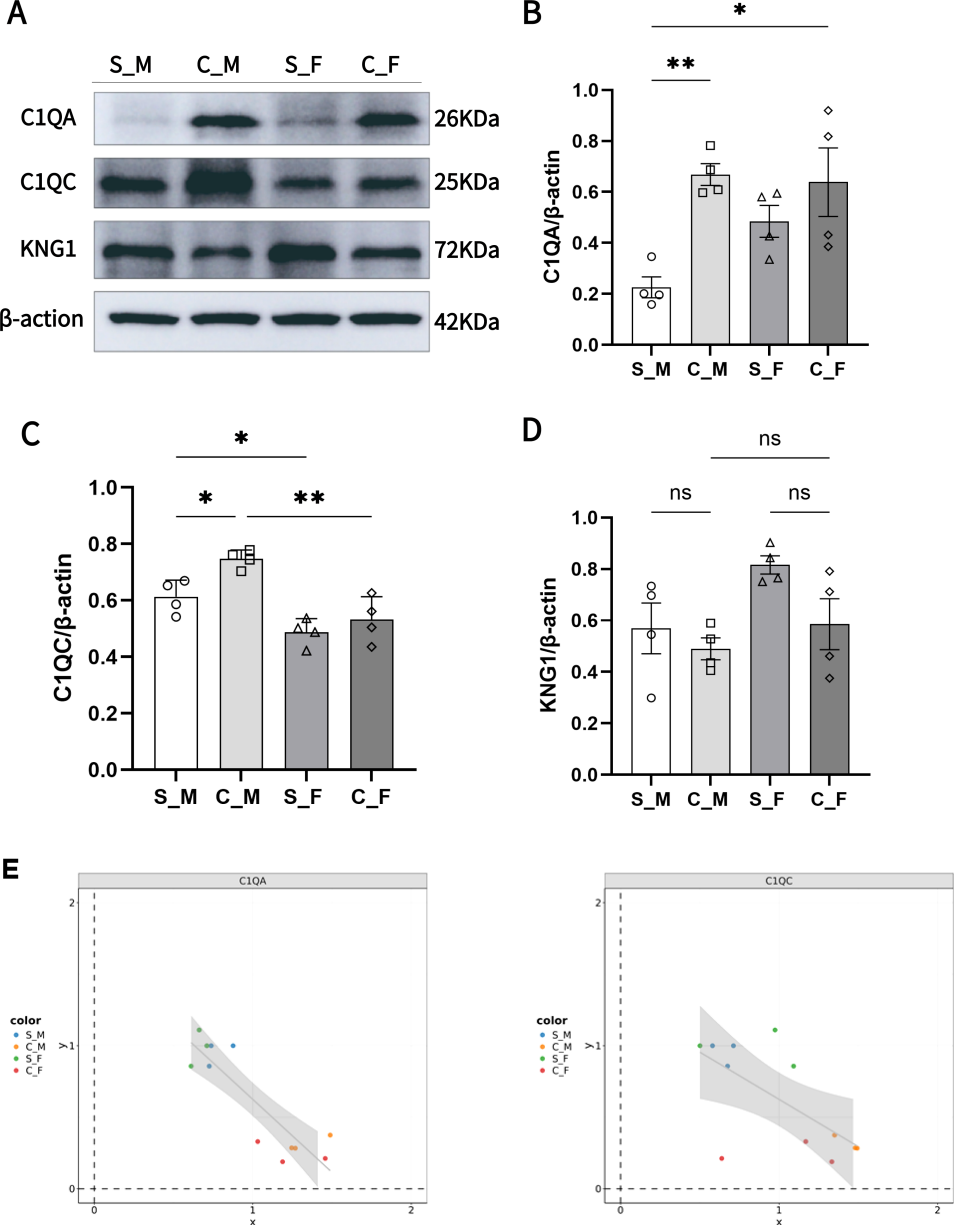
Validation of sexually dimorphic expression of C1QA, C1QC, and KNG1 and their correlation with pain behavior. **(A)** Representative Western blot images showing the expression of C1QA (26 kDa), C1QC (25 kDa), and KNG1 (72 kDa) across groups. $\beta$-actin (42 kDa) served as the loading control. **(B)** Quantification of C1QA relative expression. C1QA levels are significantly higher in female Shams (S_F) than male Shams (S_M) and are significantly upregulated in the male CION group. **(C)** Quantification of C1QC relative expression. C1QC levels are significantly higher in male Shams (S_M) than female Shams (S_F). **(D)** Quantification of KNG1 relative expression. No statistically significant differences (ns) were observed between groups in the validation cohort. **(E)** Pearson correlation analysis between the relative protein intensity (X-axis) of C1QA (left) and C1QC (right) versus the mechanical pain threshold ratio (Y-axis). The negative slope indicates that increased protein expression correlates with increased pain sensitivity (lower threshold ratio). Shaded areas represent 95% confidence intervals. Data in bar graphs are presented as mean pmSEM. Statistical significance: * p < 0.05, ** p < 0.01.

To corroborate the proteomic findings, we validated the expression of key Complement and Coagulation Cascade pathway proteins—C1QA, C1QC, and KNG1—via Western blot (WB) analysis (**Figure 5A**).

KNG1: While proteomic profiling suggested downregulation of Kininogen-1 (KNG1) in the TN model, WB validation revealed no statistically significant differences between CION and Sham groups for either sex (**Figure 5D**). However, a clear trend of sexual dimorphism was observed, with female rats exhibiting generally higher baseline KNG1 expression compared to males, consistent with the proteomic dataset.

C1q Complex: Validation of the C1q subunits revealed distinct regulatory patterns. C1QA expression was significantly elevated in the male TN group compared to controls (C_M vs. S_M, p < 0.01). A baseline sex difference was also confirmed, with female Sham rats (S_F) displaying significantly higher C1QA levels than male Sham rats (S_M, p < 0.05) (**Figure 5B**). Conversely, C1QC displayed an opposing pattern of sexual dimorphism; baseline levels were significantly higher in males (S_M) than in females (S_F, p < 0.01). In the disease model, C1QC was significantly upregulated in males (C_M vs. S_M, p < 0.05) but not in females (**Figure 5C**).

Correlation with Pain Behavior: To link molecular alterations with phenotypic outcomes, we performed a correlation analysis between protein abundance (MS intensity) and mechanical pain thresholds (expressed as the ratio of post-surgery to baseline threshold). The analysis revealed a negative correlation for both C1QA and C1QC (**Figure 5E**). Specifically, higher expression levels of these complement proteins correlated with lower pain threshold ratios, indicating that elevated C1q abundance is associated with exacerbated mechanical hypersensitivity. Collectively, these data confirm the involvement of the complement pathway in TN pathogenesis and highlight C1QA as a pivotal factor driving female susceptibility through sexually dimorphic expression.

### 3.6 Phosphoproteomic profiling highlights the role of MAPK signaling in trigeminal neuralgia pathogenesis

Protein phosphorylation is a pivotal post-translational modification governing signal transduction, playing a critical role in cellular activation and immune responses. To elucidate the signaling mechanisms underlying TN, we analyzed differentially expressed phosphoproteins (DEPPs) and their functional enrichment. Gene Ontology (GO) analysis revealed that upregulated DEPPs were significantly enriched in pathways regulating tau-protein kinase activity and calmodulin-dependent protein phosphatase activity (**Supplementary Figure 1A**). Conversely, downregulated DEPPs were primarily associated with the regulation of metal ion transport and ion homeostasis (**Supplementary Figure 1B**).

**Figure 6:**
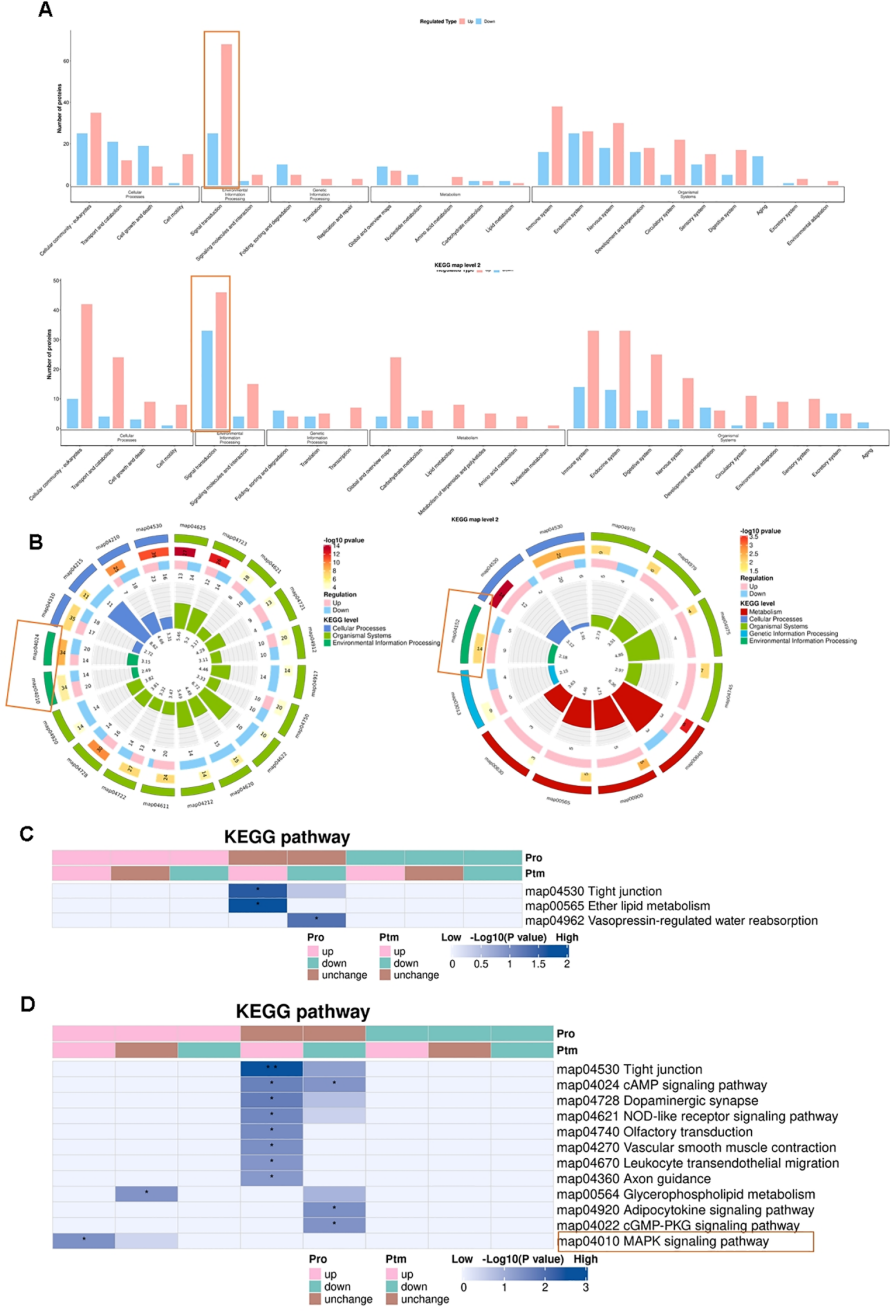
Phosphorylation-mediated regulation of MAPK signaling is a hallmark of Trigeminal Neuralgia. **(A)** KEGG functional classification of differentially expressed phosphoproteins (DEPPs). The bar charts display the number of proteins enriched in top categories for the CION/Sham comparison (top) and the S_M/S_F comparison (bottom). In both comparisons, DEPPs are predominantly concentrated in the Signal Transduction category (orange box). **(B)** Circos plots depicting the top 20 enriched KEGG pathways for CION/Sham (left) and S_M/S_F (right). The plot layers (from outside to inside) represent: (1) Enriched pathway categories; (2) Number of DEPPs and significance (p-value intensity); (3) Bar chart of upregulated (pink) and downregulated (blue) phosphoproteins; (4) Fold enrichment score. Note: Only pathways with \ge4 associated genes are displayed. **(C, D)** Integrated heatmap analysis of KEGG pathway enrichment for Proteomic (Pro) and Phosphoproteomic (PTM) datasets. The heatmaps compare enrichment significance in the S_M/S_F group **(C)** and the CION/Sham group **(D)**. Blue intensity corresponds to the significance level (-log10P). Asterisks denote statistical significance: * p < 0.05, ** p < 0.01, *** p < 0.001. The MAPK signaling pathway (orange box in D) shows significant enrichment in both datasets for the TN model.

KEGG functional classification demonstrated that DEPPs in both the TN model (CION vs. Sham) and the baseline sex-comparison (S_M vs. S_F) were predominantly clustered within the Signal Transduction category (**Figure 6A**). However, specific pathway enrichment differed markedly between these groups. In the S_M vs. S_F comparison, the AMPK signaling pathway (map04152) was the most significantly enriched (**Figures 6B, right**). In contrast, the CION vs. Sham comparison showed significant enrichment in the MAPK signaling pathway (map04010) and cAMP signaling pathway (map04024) (**Figures 6B, left**).

To distinguish between expression-level and modification-level regulation, we performed an integrated analysis of the proteome (Pro) and phosphoproteome (PTM). In the S_M vs. S_F comparison, the Tight junction pathway (map04530) exhibited significant alterations exclusively at the phosphorylation level, with no corresponding changes in total protein abundance (**Figure 6C**). In contrast, the integrated analysis of the TN model (CION vs. Sham) revealed a synchronized upregulation of the MAPK signaling pathway—specifically involving the heat shock protein HSPB1—at both the total protein and phosphorylation levels (**Figure 6D**). These findings implicate the dual regulation of MAPK signaling as a critical molecular driver in the pathogenesis of TN.

### 3.7 Activation of MAPKAPK2/3-mediated phosphorylation of HSPB1 at serine 86 in trigeminal neuralgia

**Figure 7:**
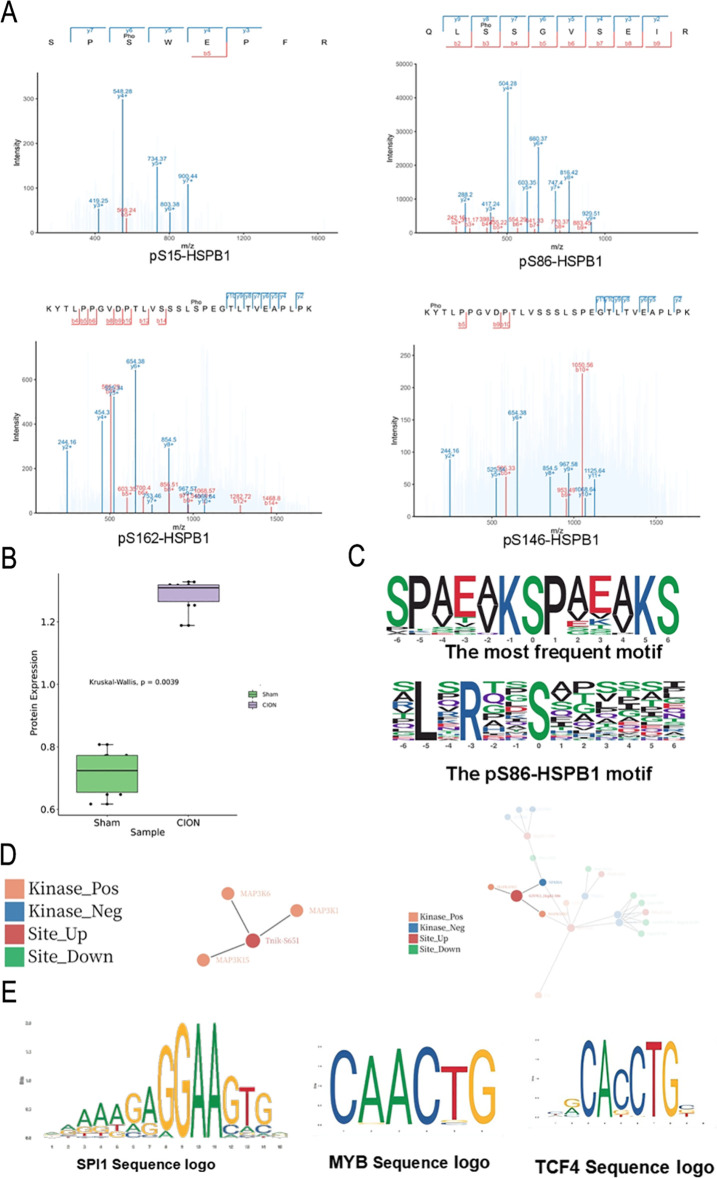
Characterization of HSPB1 phosphorylation and upstream kinase prediction**(A)** Representative mass spectrometry spectra identifying four phosphorylation sites on HSPB1: pS15, pS86, pS162, and pS146. **(B)** Quantification of pS86-HSPB1 levels in TG tissues from CION and Sham groups. The box plot indicates a significant increase in phosphorylation in the TN model. Statistical significance was determined using the Kruskal-Wallis test (p = 0.0039). **(C)** Consensus motif analysis. Top: The most frequent motif extracted from the global phosphoproteomic dataset. Bottom: The specific sequence logo surrounding the pS86-HSPB1 site. **(D)** Kinase-substrate regulatory network predicting upstream kinases for pS86-HSPB1. The network analysis identifies MAPKAPK2 and MAPKAPK3 as putative kinases regulating this site in both male (left) and female (right) TN models. **(E)** Sequence logos representing the predicted DNA binding motifs for transcription factors SPI1, MYB, and TCF4, identified as potential downstream targets in the HSPB1-associated regulatory network.

Our proteomic analysis previously identified significant upregulation of HSPB1 in the TN model (Section 3.3). To investigate the post-translational regulation of this protein, we analyzed the phosphoproteomic dataset, which identified four distinct phosphorylation sites on HSPB1: Ser15 (S15), Ser86 (S86), Ser162 (S162), and Ser146 (S146) (**Figure 7A**). Among these, phosphorylation at Serine 86 (pS86-HSPB1) was consistently detected across all samples. Quantification revealed that pS86-HSPB1 levels were significantly elevated in the TG tissues of the CION group compared to the Sham group (**Figure 7B**; Kruskal-Wallis test, p = 0.0039), mirroring the upregulation of total HSPB1 protein.

To elucidate the kinase specificity governing these modifications, we performed motif analysis on the identified phosphopeptides. Motif-X analysis extracted the most frequent amino acid sequences flanking the phosphorylation sites, revealing a specific consensus motif for the pS86-HSPB1 site (**Figure 7C**). Furthermore, Kinase-Substrate Enrichment Analysis (KSEA) indicated a distinct activation pattern of upstream kinases. The female TN group (C_F) exhibited the highest number of predicted activated kinases, followed by the male TN group (C_M) (**Supplementary Figure 3**). Notably, baseline sexual dimorphism was observed, with female Sham rats (S_F) showing higher kinase activity than male Sham rats (S_M) (**Supplementary Figure 4**).

We subsequently constructed a kinase-substrate regulatory network, which predicted that pS86-HSPB1 is a direct substrate of MAPKAPK2 and MAPKAPK3 (**Figure 7D**). Both kinases share high sequence homology and are canonical downstream effectors activated by p38 MAPK. Given that HSPB1 overexpression can repress the transactivation activity of Heat Shock Transcription Factor 1 (HSF1)—which is also regulated by MAPKAPK2—we further explored potential downstream transcriptional targets using the Animal TFDB database. We identified high-probability DNA binding motifs for transcription factors potentially associated with this pathway, including SPI1, MYB, and TCF4 (**Figure 7E** and **Supplementary Table 2**). Specifically, the analysis highlighted a high-confidence binding site for SPI1 (nucleotides 1880-1901), suggesting a complex regulatory network linking MAPK signaling, HSPB1 phosphorylation, and downstream transcriptional modulation in TN pathology.

### 3.8 Upstream regulation of MAPK signaling via voltage-gated calcium channels and therapeutic implications

Our previous GO analysis indicated a concentration of differentially expressed phosphoproteins (DEPPs) in metal ion transport pathways (**Supplementary Figure 1B**). To identify the upstream initiators of the MAPK signaling cascade in TN, we examined the phosphorylation status of membrane receptors. We observed no significant phosphorylation changes in classical immune receptors such as IL-1R, TNFR, CD14, or FAS. However, distinct alterations were identified in voltage-gated calcium channels: phosphorylation of the P/Q-type channel subunit CACNA1A was significantly downregulated, whereas the N-type channel subunit CACNA1B was significantly upregulated in both female and male TN models (**Supplementary Table 3**).

**Figure 8:**
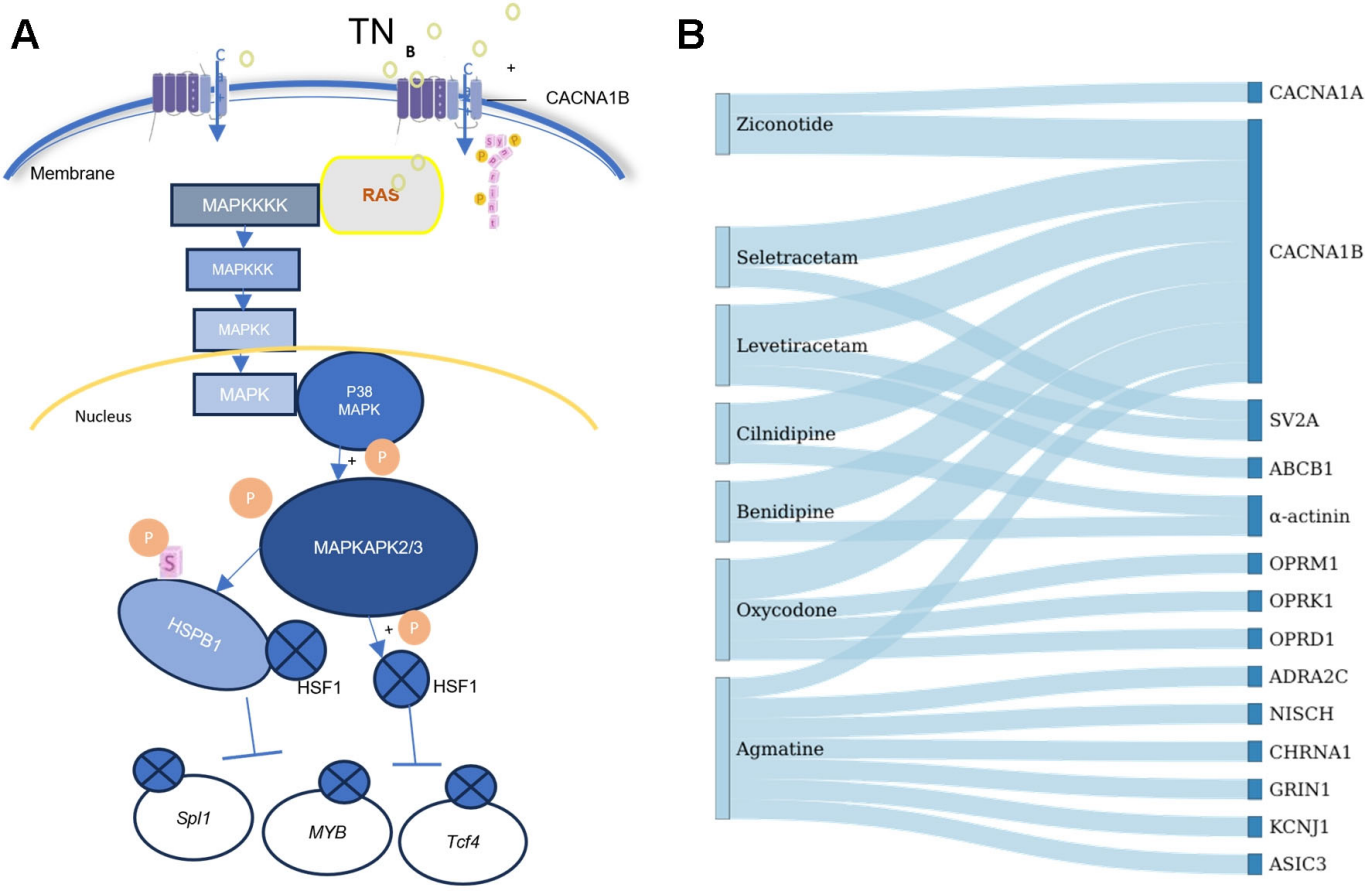
Mechanism of CACNA1B-mediated MAPK pathway activation and therapeutic targeting. **(A)** Schematic diagram of the proposed signaling pathway in Trigeminal Neuralgia (TN). TN induces the phosphorylation of the voltage-gated calcium channel subunit CACNA1B, promoting N-type calcium influx. This triggers the MAPK signaling cascade (RAS rightarrow MAPK rightarrow p38 MAPK), leading to the activation of MAPKAPK2/3. Activated MAPKAPK2/3 phosphorylates HSPB1 (at pS86), which subsequently inhibits the transcription factor HSF1, preventing its binding to downstream DNA targets (Spi1, Myb, Tcf4). **(B)** Sankey diagram illustrating drug-target interactions for calcium channels. Clinical drugs (left) are mapped to their molecular targets (right). The flow width represents the association. Ziconotide is highlighted for its specificity to calcium channels (CACNA1B/CACNA1A) compared to other agents like Levetiracetam or Cilnidipine which target broader protein families.

These findings suggest that TN promotes the phosphorylation of the transmembrane glycoprotein CACNA1B, facilitating N-type calcium influx. This calcium signaling event likely acts as the upstream trigger for the intracellular MAPK cascade, leading to the activation of p38 MAPK and subsequently MAPKAPK2/3. As detailed in Section 3.7, activated MAPKAPK2/3 phosphorylates HSPB1 at Ser86. This phosphorylation event modulates the conformational state of the Heat Shock Transcription Factor 1 (HSF1), thereby inhibiting its binding to downstream target genes such as Spi1, Myb, and Tcf4 (**Figure 8A**).

Given the pivotal role of CACNA1B in initiating this pathological cascade, we evaluated its potential as a therapeutic target. DrugBank database analysis mapped several clinical agents to N-type calcium channels, including antiepileptics (e.g., Levetiracetam) and antihypertensives (e.g., Cilnidipine). Notably, the analysis highlighted Ziconotide as a highly specific agent, targeting CACNA1B and CACNA1A without the broader off-target interactions observed with other agents (**Figure 8B**). Ziconotide is currently the only FDA-approved calcium channel-blocking peptide indicated for the management of severe, refractory chronic pain, validating the clinical rationale for targeting the CACNA1B-MAPK signaling axis in trigeminal neuralgia.

## 4 Discussion

Trigeminal neuralgia (TN), or tic douloureux, is a debilitating neuropathic disorder characterized by paroxysmal, electric-shock-like pain restricted to the somatosensory distribution of the trigeminal nerve (1, 18). As a chronic condition marked by unpredictable remissions and recurrences, TN profoundly compromises functional capacity and quality of life (19). Current pharmacological standards, primarily carbamazepine, remain suboptimal; approximately two-thirds of patients continue to suffer from moderate-to-severe pain, and a significant proportion discontinue therapy due to intolerable adverse effects or the necessity for complex polypharmacy (20). Consequently, elucidating the precise molecular mechanisms driving TN is imperative for developing more effective, targeted therapeutics. While aberrant protein phosphorylation is increasingly recognized as a critical driver of neuropathic pain pathogenesis (8, 10), the specific phosphoproteomic landscape within the trigeminal ganglion (TG) during TN remains largely unexplored. This study addresses this knowledge gap by employing a modified intraoral chronic constriction injury of the infraorbital nerve (CION) model. This refined surgical approach minimizes postoperative infection risks and obviates the need for antibiotics, thereby preserving the integrity of the tissue for high-sensitivity omics analysis (21). By integrating high-throughput proteomics with phosphoproteomics (11, 13), we provide the first comprehensive map of the signaling alterations in the TG of male and female rats. Our findings not only clarify the neuroimmune interplay underlying TN but also delineate distinct sex-specific regulatory patterns, offering novel insights into the sexual dimorphism observed in clinical chronic pain states (4).

### 4.1 Activation of the complement and coagulation cascades: A core node in neuroimmune crosstalk

The most salient finding of this study is the identification of Complement C1q subcomponent subunit A (C1QA) as a pivotal driver of neuroimmune dysregulation in TN. While the complement system is a well-established component of innate immunity, the role of C1QA within the peripheral nervous system has remained underappreciated. Our data demonstrate that C1QA is not only significantly upregulated in both male and female TN models but also exhibits distinct, sex-specific expression patterns. This molecular signature mirrors the well-documented epidemiological disparity in TN incidence, where females predominate by a ratio of approximately 1.5–2:1 (22, 23). Proteomic profiling revealed a 1.8-fold increase in C1QA expression in female CION rats and a 1.2-fold increase in males compared to their respective sham controls, a finding robustly validated by Western blot analysis. As one of only eight differentially expressed proteins (DEPs) conserved across sexes, C1QA emerges as a fundamental pathogenic marker. However, the greater magnitude of upregulation in females suggests it may serve as a molecular substrate for the heightened susceptibility observed in women (22, 24). Mechanistically, C1QA-driven complement activation is likely to amplify neuroinflammation within the trigeminal ganglion (TG) by triggering microglial activation and the release of pro-inflammatory cytokines. This inflammatory milieu is known to enhance neuronal hyperexcitability, the physiological hallmark of TN (12, 25). Furthermore, our analysis reveals that C1QA functions within an integrated network involving coagulation factors and Kininogen-1 (KNG1) (Section 3.4). This interplay links complement activation with the kinin-mediated nociceptive system, establishing a critical axis of neuroimmune crosstalk that perpetuates pain signaling.

### 4.2 The KNG1-C1QA-C1QC axis: A molecular basis for sexual dimorphism in TN

Our multi-omics integration highlights the KNG1-C1QA-C1QC regulatory axis as a primary mechanism underlying the sexual dimorphism of TN. The distinct molecular landscapes of male and female rats were underscored by the minimal overlap (9.2%) in DEPs between sexes, pointing to divergent adaptive responses to nerve injury driven largely by dysregulation of the complement-kinin system (5, 24). Three key components define this sexually dimorphic axis: 1. KNG1 (Pro-nociceptive Priming): Female rats exhibited a 2.1-fold higher baseline expression of KNG1 compared to males (4). As the precursor to bradykinin—a potent sensitizer of trigeminal nociceptors (21)—this elevated baseline suggests a constitutively “primed” pro-nociceptive state in females. Although KNG1 was downregulated post-injury (likely a compensatory mechanism), absolute levels remained higher in females, potentially sustaining sensitization. 2. C1QA (Inflammatory Amplification): Consistent with clinical incidence, C1QA upregulation was markedly more pronounced in females (1.8-fold) than in males (1.2-fold). This differential expression likely drives a more aggressive neuroinflammatory response in the female TG (12, 25). 3. C1QC (Failed Regulation): We observed a sex-dependent inverse correlation between C1QA and its regulatory subunit, C1QC (26). While females possessed higher baseline C1QC (potentially to constrain complement activity), they failed to mount a significant upregulation (1.1-fold) in response to injury. In contrast, males exhibited a robust C1QC response (1.4-fold), which may buffer the pro-inflammatory effects of C1QA (27). This dysregulation is further corroborated by phosphoproteomic data, where Kinase-Substrate Enrichment Analysis (KSEA) identified a higher burden of activated kinases in females across both sham and disease groups. These molecular disparities are likely governed by interconnected biological mechanisms, including: (1) Hormonal Modulation, where estrogen enhances complement and KNG1 expression while testosterone suppresses kinase activation (24); (2) X-linked Immune Regulation, as incomplete X-inactivation may result in higher dosage of X-linked immune genes in females (4); and (3) Epigenetic Modification, where lower promoter methylation of C1QA in females facilitates its transcriptional hyperexcitability (26).

### 4.3 The MAPK-HSPB1 axis: Bridging calcium signaling and neuroinflammation

Beyond the complement system, our phosphoproteomic analysis identified the MAPK-HSPB1 signaling axis as a critical bridge connecting upstream calcium influx to downstream neuroinflammation. The enrichment of differentially expressed phosphoproteins (DEPPs) in signal transduction pathways, particularly the MAPK pathway (map04010), aligns with established pain processing networks (8, 9). A standout finding was the hyperphosphorylation of Heat Shock Protein Beta-1 (HSPB1) at Serine 86 (pS86-HSPB1), which was elevated 1.9-fold in TN models. We identified MAPKAPK2/3 as the upstream kinases responsible for this modification, consistent with their role in neurodegenerative stress responses (28). The hyperphosphorylation of HSPB1 is functionally significant; phosphorylated HSPB1 can be released extracellularly to activate microglia, thereby creating a feed-forward loop of neuroinflammation and pain chronification (28). Crucially, this pathway appears to be gated by calcium signaling via Voltage-gated Calcium Channel Subunit Alpha1 B (CACNA1B). Following nerve injury, phosphorylation of CACNA1B (N-type calcium channel) was upregulated 2.3-fold in females compared to 1.6-fold in males. Given that CACNA1B mediates nociceptive calcium influx (29, 30), this prominent phosphorylation in females likely facilitates greater calcium entry, robust activation of the MAPK cascade, and subsequent phosphorylation of HSPB1. This establishes a “Calcium-MAPK-HSPB1” axis where calcium dysregulation acts as the proximal trigger for the inflammatory cascade, contributing to the greater pain severity often observed in female subjects (24). The therapeutic relevance of this axis is substantial. Ziconotide, an FDA-approved N-type calcium channel blocker, specifically targets CACNA1B (30). By inhibiting the upstream calcium influx, Ziconotide has the potential to dampen the entire downstream MAPK-HSPB1 inflammatory cascade. We propose that validating Ziconotide in this specific context—assessing its ability to reduce pS86-HSPB1 levels and complement activation in the TG—represents a high-priority direction for translational research.

### 4.4 Therapeutic implications, limitations, and conclusion

Our integrated omics analysis delineates a complex phosphorylation network governing TN pathogenesis, highlighting the Complement-Kinin system and the MAPK-HSPB1-CACNA1B axis as actionable therapeutic targets. Importantly, the sex-specific nature of these pathways necessitates a shift toward sex-stratified precision medicine.

1. Complement Modulation: Targeting C1QA or complement receptors could attenuate neuroimmune activation. Given the higher complement baseline in females, dosing strategies may need to be adjusted by sex.
2. Disrupting the Kinase Axis: Small-molecule inhibitors of MAPKAPK2/3 or blockers of HSPB1 phosphorylation could interrupt the neuroinflammatory feedback loop.
3. Calcium Channel Blockade: Ziconotide represents a promising candidate for repurposing in TN. Its efficacy may be particularly pronounced in female patients, who exhibit higher pathological activation of CACNA1B.

Limitations: While this study provides a comprehensive molecular map, limitations exist. First, rodent models may not fully recapitulate the complexity of human TN pathophysiology (5, 6). Second, while we infer microglial activation via C1QA and HSPB1, the precise neuron-glia signaling dynamics require confirmation through spatial transcriptomics or co-culture systems (12, 14). Third, direct causality remains to be established via *in vivo* gene editing of specific phosphorylation sites. Finally, the specific hormonal contributions to C1QA/KNG1 regulation warrant detailed investigation via gonadectomy studies.

Conclusion: In summary, this study utilizes integrated proteomics and phosphoproteomics to systematically elucidate the neuroimmune landscape of Trigeminal Neuralgia. We identify the C1QA-driven Complement and Coagulation Cascades pathway as a core neuroimmune node, with KNG1, C1QA, and C1QC forming a sex-dimorphic regulatory axis that provides a molecular basis for the female-predominant incidence of TN. Furthermore, we characterize a MAPK-HSPB1-CACNA1B axis that links calcium channel dysregulation to neuroinflammation, aligning with established mechanisms of pain chronification. These findings not only advance our understanding of TN pathogenesis but also provide a robust rationale for developing sex-adapted immuno-analgesic strategies, addressing a critical unmet need in pain management.

## 5 Conclusion

This study employs an integrated proteomic and phosphoproteomic approach to systematically elucidate the molecular landscape governing neuroimmune interactions in Trigeminal Neuralgia (TN). Our findings implicate C1QA-driven complement activation and the CACNA1B-MAPK-HSPB1 phosphorylation axis as critical mechanisms underlying the pathogenesis and progression of TN. Specifically, the sexual dimorphism observed in the KNG1-C1QA-C1QC regulatory axis offers a plausible molecular basis for the epidemiological prevalence of TN in females. These insights not only advance our understanding of the complex neuroimmune crosstalk in neuropathic pain but also provide a theoretical rationale for developing sex-stratified, targeted immuno-analgesic therapies. Future investigations should focus on validating these targets in clinical TN cohorts and exploring the efficacy of integrating neuroimmune modulation, such as calcium channel blockade or complement inhibition, into comprehensive pain management protocols.

## Data Availability

The datasets presented in this study can be found in online repositories. The names of the repository/repositories and accession number(s) can be found below: http://www.proteomexchange.org/, PXD066745.
