# Supplementary material for: Phosphoproteomics uncovers a neuroimmune perspective on trigeminal neuralgia: sexually dimorphic regulatory networks linking calcium channels to the complement cascade
Source: Front Immunol. 2026 Mar 3;17:1676019. doi: 10.3389/fimmu.2026.1676019 (PMC12992019; doi:10.3389/fimmu.2026.1676019)
Supplement: Supplementary file 1 [file DataSheet1.docx]

**Table S1 The types of changes in Gap43 across different comparison groups**

| **Compared sample name** | **Protein accession** | **Gene name** | **Fold changes** | **P value** | **Regulated Type** |
| --- | --- | --- | --- | --- | --- |
| **C_F/S_F** | P07936 | Gap43 | 2.3897917 | 0.0039248 | Up |
| **C_M/S_M** | P07936 | Gap43 | 1.664224 | 0.0316786 | Up |
| **S_F/S_M** | P07936 | Gap43 | 1.6132955 | 0.0171427 | Up |
| **C_F/C_M** | P07936 | Gap44 | 0.3497267 | 1.664224 | Unchange |

**If the fold change is greater than 1.5 and the p-value is less than 0.05, we consider it to be significantly upregulated; otherwise, it is deemed invalid.**

**Table S2. The regulated type of pS86-HSPB1 across different groups**

| **Compared sample name** | **Modification site** | **Fold changes** | **P value** | **Regulated Type** |
| --- | --- | --- | --- | --- |
| **C_F/S_F** | pS86-HSPB1 | 1.8209835 | 0.0009671 | Up |
| **C_M/S_M** | pS86-HSPB1 | 1.7695347 | 0.002213 | Up |
| **S_M/S_F** | pS86-HSPB1 | 1.0140599 | 0.9024768 | Unchange |
| **C_F/C_M** | pS86-HSPB1 | 1.0148067 | 0.7056811 | Unchange |

**If the fold change is greater than 1.5 and the p-value is less than 0.05, we consider it to be significantly upregulated; otherwise, it is deemed invalid.**

**Table S3. Regulated type of two Calcium channels**

| **Type** | **Group** | **Regulated type** |
| --- | --- | --- |
| **CACN1A** | C_M/S_M | Down |
| **CACN1B** | C_F/S_F | Up |
| **CACN1B** | C_M/S_M | Up |

**Table S4. Prediction of HSF1 top five Transcription Factor Binding Site (TFBS)**

| **TFBS** | **Start** | **Stop** | **Stand** | **Score** | **P-Value** | **Q-Value** |
| --- | --- | --- | --- | --- | --- | --- |
| Spl1 | 1880 | 1901 | - | 19.8714 | 1.25E-07 | 0.00044900 |
| MYB | 1806 | 1830 | + | 19.8684 | 1.03E-07 | 0.00031300 |
| Tcf4 | 1640 | 1661 | - | 19.7237 | 1.43E-07 | 0.00041900 |
| Sp1 | 1882 | 1903 | - | 19.4394 | 1.66E-07 | 0.00058900 |
| Spi1 | 1881 | 1902 | + | 19.3857 | 1.56E-07 | 0.00059500 |

**(A)**


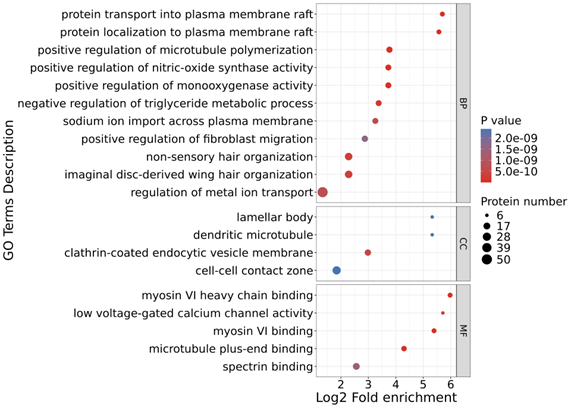

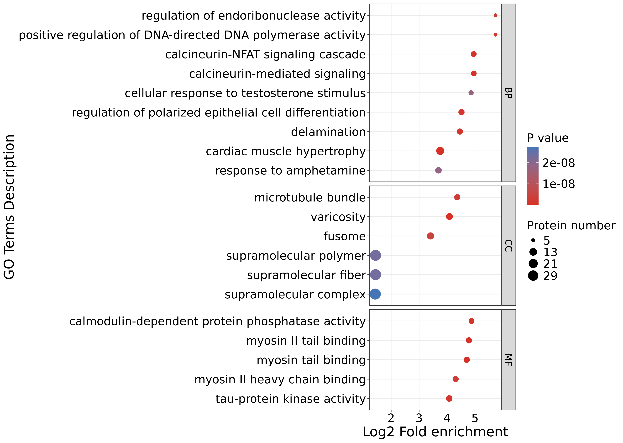


**(B)**

**Figure S1. Gene Ontology (GO) functional annotation of differentially expressed phosphoproteins (DEPPs).**

**(A) Bubble chart illustrating the GO enrichment of upregulated DEPPs. Key enriched biological processes (BP) and molecular functions (MF) include tau-protein kinase activity and phosphatase signaling. (B) Bubble chart illustrating the GO enrichment of downregulated DEPPs, highlighting pathways associated with metal ion transport and membrane organization. The size of each bubble represents the number of proteins, and the color gradient represents the p-value significance.**


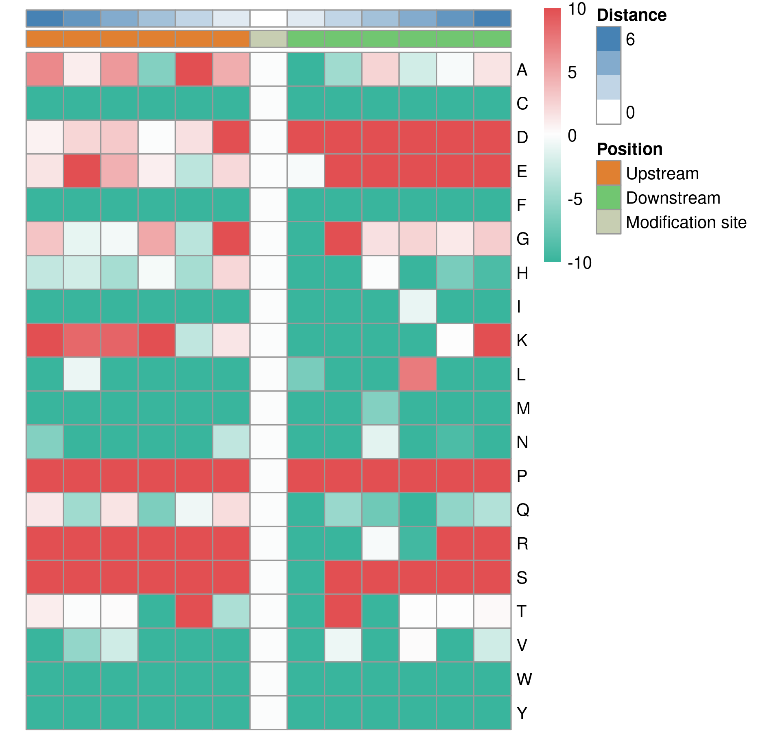


**Figure S2. Motif analysis of phosphorylation modification**

**Figure S3. Number of protein kinases between different groups**


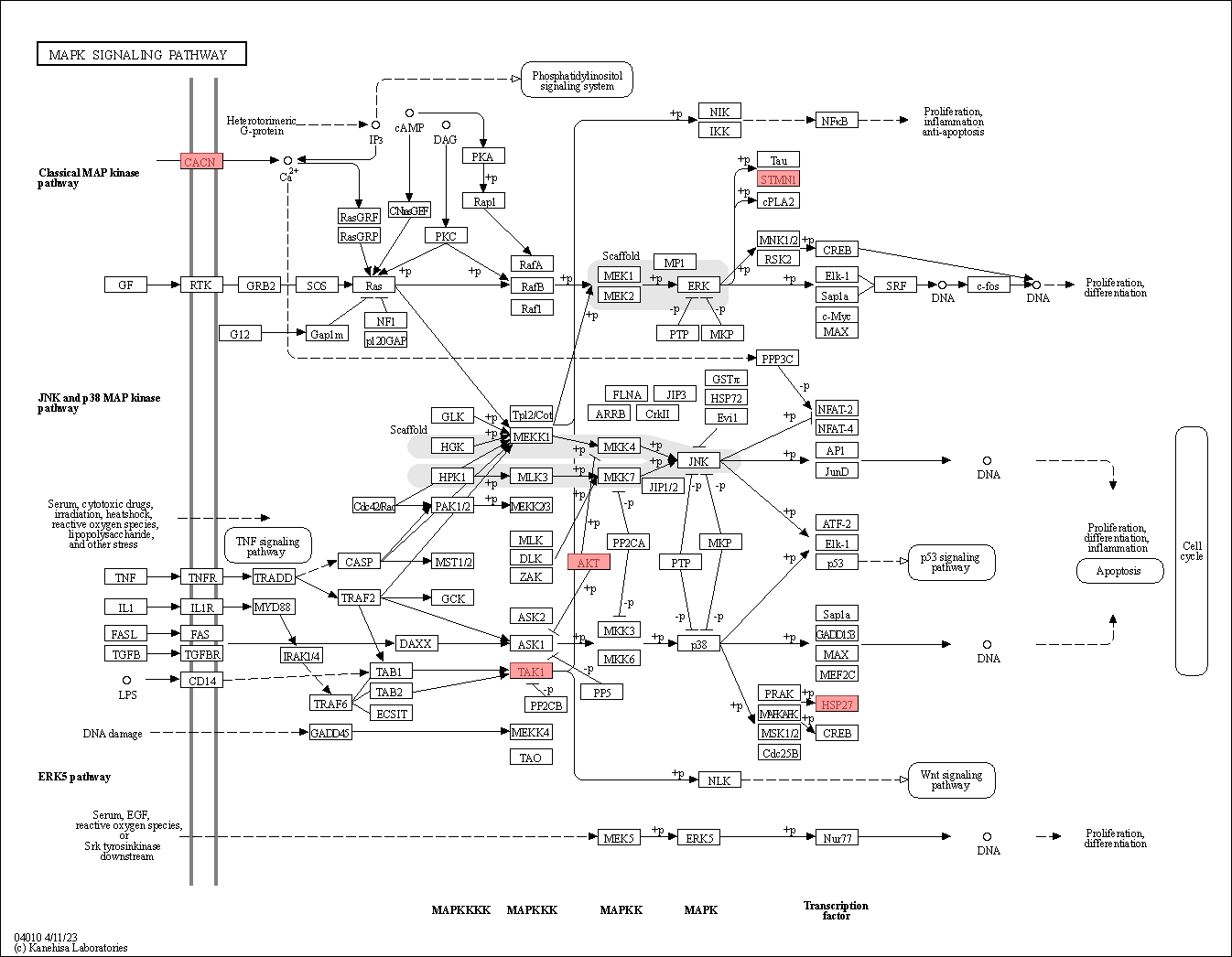


（B）

(A)


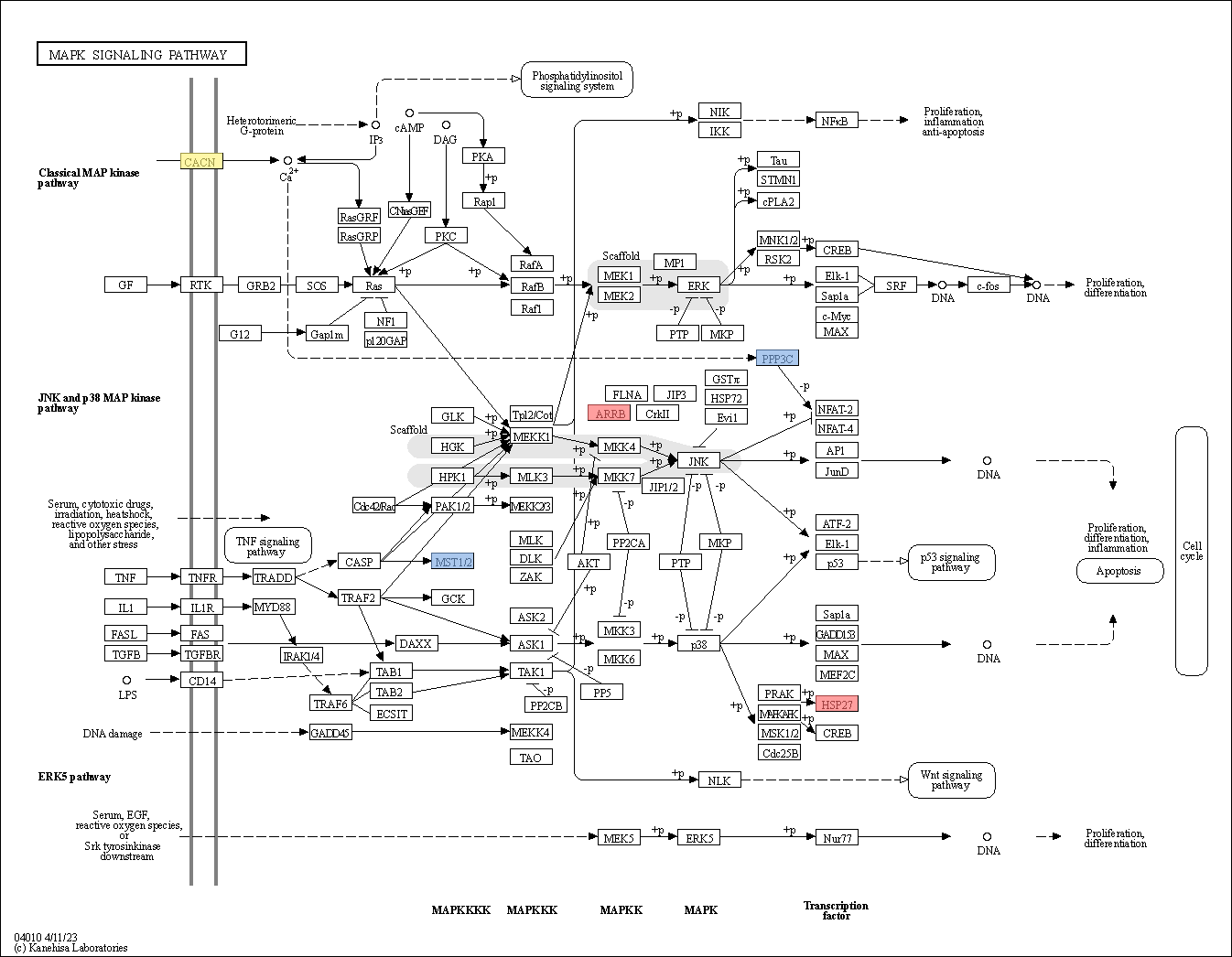


**Figure S4. The MAPK signaling pathway differentially expressed phosphoproteins (DEPPs)**

(A)The differentially expressed phosphoproteins (DEPPs) in C_F/S_F and (B) C_M/S_M. Red represents upregulated proteins, blue represents downregulated proteins, and yellow represents both upregulated and downregulated proteins.


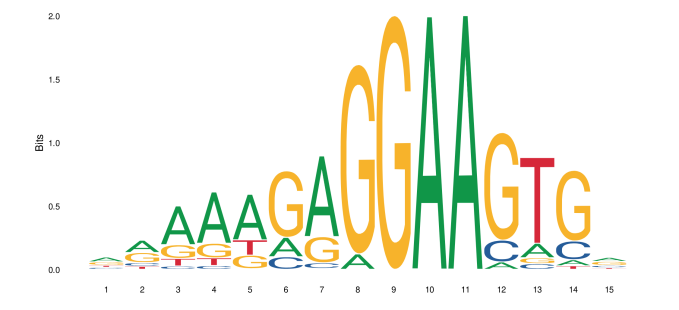


**SPI1 Sequence logo**


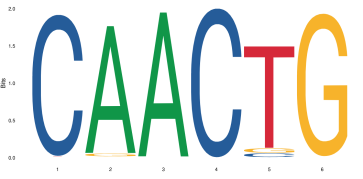


**MYB Sequence logo**


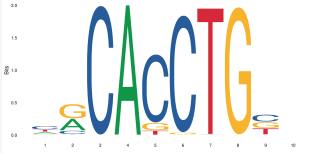


**TCF4 Sequence logo**

**Figure S5** **Top Predicted HSF1-Binding Site and Other TFBS Sequences**
